# COSMOS-E: Guidance on conducting systematic reviews and meta-analyses of observational studies of etiology

**DOI:** 10.1371/journal.pmed.1002742

**Published:** 2019-02-21

**Authors:** Olaf M. Dekkers, Jan P. Vandenbroucke, Myriam Cevallos, Andrew G. Renehan, Douglas G. Altman, Matthias Egger

**Affiliations:** 1 Department of Clinical Epidemiology, Leiden University Medical Center, Leiden, the Netherlands; 2 Department of Clinical Endocrinology and Metabolism, Leiden University Medical Centre, Leiden, the Netherlands; 3 Department of Clinical Epidemiology, Aarhus University Hospital, Aarhus, Denmark; 4 Faculty of Epidemiology and Population Health, London School of Hygiene and Tropical Medicine, London, United Kingdom; 5 Institute of Social and Preventive Medicine (ISPM), University of Bern, Bern, Switzerland; 6 Manchester Cancer Research Centre, NIHR Manchester Biomedical Research Centre, Division of Cancer Sciences, School of Medical Sciences, Faculty of Biology, Medicine and Health, University of Manchester, Manchester, United Kingdom; 7 Centre for Statistics in Medicine, Nuffield Department of Orthopaedics, Rheumatology and Musculoskeletal Sciences, University of Oxford, Oxford, United Kingdom; 8 Centre for Infectious Diseases Epidemiology and Research (CIDER), School of Public Health and Family Medicine, University of Cape Town, Cape Town, South Africa

## Abstract

**Background:**

To our knowledge, no publication providing overarching guidance on the conduct of systematic reviews of observational studies of etiology exists.

**Methods and findings:**

Conducting Systematic Reviews and Meta-Analyses of Observational Studies of Etiology (COSMOS-E) provides guidance on all steps in systematic reviews of observational studies of etiology, from shaping the research question, defining exposure and outcomes, to assessing the risk of bias and statistical analysis. The writing group included researchers experienced in meta-analyses and observational studies of etiology. Standard peer-review was performed. While the structure of systematic reviews of observational studies on etiology may be similar to that for systematic reviews of randomised controlled trials, there are specific tasks within each component that differ. Examples include assessment for confounding, selection bias, and information bias. In systematic reviews of observational studies of etiology, combining studies in meta-analysis may lead to more precise estimates, but such greater precision does not automatically remedy potential bias. Thorough exploration of sources of heterogeneity is key when assessing the validity of estimates and causality.

**Conclusion:**

As many reviews of observational studies on etiology are being performed, this document may provide researchers with guidance on how to conduct and analyse such reviews.

## Abstract 2

In a Guidelines and Guidance article, Olaf Dekkers and colleagues discuss COSMOS-E, in which they aim to provide guidance for those performing systematic reviews and meta-analyses of observational studies addressing etiology.

## Introduction

Systematic reviews aim to appraise and synthesise the available evidence addressing a specific research question; a meta-analysis is a statistical summary of the results from relevant studies. A systematic review should generally be the basis of a meta-analysis, whereas a meta-analysis is not a necessary feature of a systematic review if reviewers decide that pooling of effect estimates is not appropriate. Many systematic reviews are based on observational studies. In 2014, of about 8,000 systematic reviews published that year, 36% were on etiology, prognosis, or diagnosis [1].

Etiological studies examine the association of exposures with diseases or health-related outcomes. Exposures potentially causing diseases are also called risk factors and may take many forms; they can be fixed states (e.g., sex, genetic factors) or vary over time, for example metabolic risk factors (e.g., hypercholesterolemia, insulin resistance, hypertension), lifestyle habits (e.g., smoking, diet), or environmental factors (e.g., air pollution, heat waves). Conceptually, these exposures differ from interventions, which explicitly aim to influence health outcomes and have a clear starting point in time [2]. Observational studies are important to study exposures that are difficult or impossible to study in randomised controlled trials (RCTs), such as air pollution or smoking. Also, observational studies are important to study causes with long latency time, such as carcinogenic effects of environmental exposures or drugs.

The epidemiological study of risk factors typically relies on comparisons (exposed versus unexposed); such comparisons can be made in cohort studies in which exposed and unexposed people are followed over time [3]. Other approaches such as self-controlled studies, case-control studies, cross-sectional studies, ecological studies, instrumental variable analyses, and mendelian randomisation also rely on comparisons. Box 1 presents an overview of observational study designs used to study etiology.

**Box 1. Observational designs and approaches for studying etiology**

Cohort study

Cohort studies follow a study population over time. Researchers can study the occurrence of different outcomes. In etiological research, an exposed and an unexposed group are compared regarding the risk of the outcome. Different levels of exposure and exposures that vary over time can be studied. Instrumental variable methods and self-controlled case series studies are types of cohort studies (see below).

Example

In a large population-based cohort study, the occurrence of infectious complications was compared between patients with and patients without Cushing disease [4].

Instrumental variable methods/mendelian randomisation

Instrumental variable (IV) analyses use an external factor that determines the exposure of interest but is (ideally) not associated with the outcome other than through its effect on the exposure. In other words, the instrument is not associated with the factors that may confound the association between exposure and outcome. The instrument can be calendar time, geographical area, or treatment preferences [5,6]. Mendelian randomisation studies are examples of IV analyses using genetic factors as instruments.

Example

A Mendelian randomisation study investigated whether more years spent in education increase the risk of myopia or whether myopia leads to more years spent in education [7].

Self-controlled designs

In self-controlled case series, the occurrence of the outcome is compared between time windows during which individuals are exposed to a risk factor and time windows not exposed. In contrast to standard cohort designs, the comparison is within individuals. The design is used to study transient exposures for which exact timings are available, such as infections, vaccinations, drug treatments, climatic exposures, or disease exacerbations [8].

Example

A self-controlled study examined the effect of cold spells and heat waves on admissions for coronary heart disease, stroke, or heart failure in Catalonia [9].

Case-control study

In case-control studies, exposures are compared between people with the outcome of interest (cases) and people without (controls) [3]. The design is especially efficient for rare outcomes.

Example

A multicentre case-control study examined the association between mobile phone use and primary central nervous system tumours (gliomas and meningiomas) in adults [10].

Cross-sectional studies

In cross-sectional studies, study participants are assessed at the same point in time to examine the prevalence of exposures, risk factors, or disease. The prevalence of disease is then compared between exposure groups like in a cohort study, or the odds of exposure are compared between groups with and without disease, like in a case-control study [3]. The temporal relationship between exposure and outcome can often not be determined in cross-sectional studies.

Example

A cross-sectional analysis of the United Kingdom Biobank study examined whether neighbourhood exposure to fast-food outlets and physical activity facilities was associated with adiposity [11].

Ecological studies

In ecological studies, the association between an exposure and an outcome is studied and compared between populations that differ geographically or in calendar time. Limitations include the ecological fallacy, in which associations observed at the aggregate level do not hold at the individual level and confounding, which is often difficult to control.

Example

An ecological study of male circumcision practices in different regions of sub-Saharan Africa and HIV infection found that HIV prevalence was lower in areas where male circumcision was practiced than in areas where it was not [12]. The protective effect of male circumcision was later confirmed in randomised trials [13].

## Aim and scope of COSMOS-E

For systematic reviews of randomised trials, guidelines on conduct [14] and reporting [15] have been widely adopted. For systematic reviews of observational studies, reporting guidelines were published almost two decades ago [16], and, to date, there are, to our knowledge, no guidelines on their conduct. Despite similarities in the general structure of the review, the ‘roadmap’ of systematic reviews of observational studies is less standardised [17], and some design issues are not settled yet [18]. The aim of Conducting Systematic Reviews and Meta-Analyses of Observational Studies of Etiology (COSMOS-E) is to discuss and give guidance on key issues in systematic reviews of observational studies of etiology for researchers. We address all steps in a review on observational studies, even though some will be similar to reviews of RCTs of medical interventions. COSMOS-E covers the basic principles as well as some more advanced topics but does not address systematic reviews of nonrandomised studies of interventions, or of diagnostic, prognostic, or genetic studies. COSMOS-E is not formally meant to be a guideline; it provides guidance but does not formally prescribe how researchers should perform or report their review. Also, this paper will not settle some ongoing debates and controversies [18] around the performance of reviews of observational studies on etiology, but rather will give the different viewpoints and possibilities. The writing group included researchers experienced in meta-analyses and observational studies of etiology. No external advise was sought; standard peer-review was performed.

## Preparing the systematic review

### Building a review team

At the design stage, the team should cover both content knowledge and methodological expertise. For example, identifying potential confounding variables or assessing exposure measurements requires content knowledge. Similarly, the statistical analysis can often be complex, highlighting the need for statistical expertise. Questions may arise about whether and how different designs (for example, case-control studies and cohort studies) can be combined in meta-analysis or whether a dose-response meta-analysis is feasible. An information specialist will ensure comprehensive and efficient literature searches.

### Shaping the research question

A systematic review of observational studies requires a clear research question, which can be broad initially but should be narrowed down subsequently in the interest of clarity and feasibility. In other words, and contrasting with systematic reviews for RCTs, the research question may be iterative. After framing the question, reviewers should scope key papers to get an idea of what evidence is available about the problem, including what type of research has been done. This exploratory step has two aims. It clarifies whether the question has already been addressed in a recent systematic review and indicates whether and how the question needs to be refined and focused so that it can be the subject of feasible systematic review.

### Defining population, exposure, contrast, and outcome

In line with the well-known Population, Intervention, Control, and Outcome(s) (PICO) format [15], for systematic reviews of epidemiological studies, Population, Exposure, Control, and Outcome(s) (PECO) should be defined [19]. The study population should reflect the target population, i.e., the population to which the results should be applicable [20]. This can be the general population, as in a meta-analysis of the association of insulin-like growth factor and mortality [21], or a restricted population, such as in a review of the association between breastfeeding and childhood leukemia [22]. The study population must be defined such that the exposure–outcome association can be validly studied [23]. For example, if a radiation exposure is assumed to damage growing tissues, then children are a more appropriate study population than adults (see discussion of ‘study sensitivity’ [23] later in this article).

Studies on risk factors should ideally include people who are free of the outcome under study at start of follow-up, but this is often unverifiable in population-based studies. Subclinical or early disease might go undetected if such conditions are not ruled out explicitly. In a review on the association between insulin resistance and cardiovascular events, not all population-based studies explicitly excluded participants with cardiovascular disease at baseline. These studies were not excluded but considered at higher risk of bias [24].

Exposure(s) and outcome(s) should be clearly defined. The definition and measurement of many exposures in observational studies of etiology, such as socioeconomic status, diet, exercise, or environmental chemicals, need careful attention, and the comparability of assessments across studies needs to be assessed. Similarly, many outcomes—such as diseases (e.g., breast cancer, thrombosis, diabetes mellitus) or health-related states (e.g., quality of life, levels of risk factors)—can be defined, classified, or measured in many different ways. Consideration of outcomes thus includes not only ‘what’ is the outcome of interest but also ‘how’ it was determined. Only death is insensitive to method of measurement or ascertainment. The exposure–control comparison also needs attention. In a study of the effect of leisure physical activity, the exposure category (for example, weekly sport for more than 2 hours) might be compared to either less than 2 hours per week or to no sport. Neither of these comparisons is wrong, but they address different questions.

Reviewers may develop precise definitions and criteria for exposure and outcomes, but if no single study used these definitions, the review will not be able to answer the research question. Iteration and pragmatism are required in this situation. In the case of fat mass and cardiovascular risk, a sophisticated exposure measurement—magnetic resonance imaging (MRI)—may have been used only in a few small studies. The reviewers may then decide also to include studies using body mass index or waist:hip ratio, which will ensure the inclusion of more and larger studies. Whether the studies can be combined statistically in a meta-analysis is a different question, which we will address later in this article.

### Considering confounding and bias

Confounding is a crucial threat to the validity of observational studies. Confounding occurs when comparison groups differ with respect to their risk of the outcome beyond the exposure(s) of interest due to a common cause of exposure and outcome. When planning the review, researchers should carefully consider which factors might potentially confound the exposure–outcome associations under study. Importantly, confounding is not a yes/no phenomenon but a matter of degree. For example, strong confounding is expected in studies that compare mortality between vegetarians and nonvegetarians because these groups will differ in many other lifestyle characteristics, which will be associated with causes of death. The opposite is true when studying smoking as a cause of lung cancer. There will be little confounding because other strong risk factors for lung cancer are rare, and it is also unlikely that they are strongly associated with smoking. In general, strong confounding is unlikely for adverse effects that were unexpected at the time the study was conducted, for example, the link between asbestos and mesothelioma [25]. Indeed, substantial confounding may be rare in occupational epidemiology, even by risk factors strongly associated with the outcome of interest [26].

Other threads to the validity of the effect estimation are measurement (misclassification) bias or selection bias. Misclassification is a crucial bias in environmental and occupational epidemiology, particularly for long-term exposures [26]. Thinking up front about potential confounding and bias will facilitate the ‘scoping’ of the crucial validity threats for the specific research question and judgments on what types of studies are likely to provide the most unbiased estimate.

### The protocol

Every systematic review should be planned in a detailed protocol. The key issues that need to be addressed are listed in Box 2. It is not always possible to specify fully all review methods beforehand; the writing of the study protocol will often be an iterative process, informed by scoping the literature and piloting procedures. Reviewers should take care not to change the protocol based on study results, but the protocol may be adapted, for example, based on the number and type of available studies. Registering the protocol in the International Prospective Register of Systematic Reviews (PROSPERO) [27] increases transparency and allows editors, peer reviewers, and others to compare planned methods with the published report and to identify inconsistencies or selective reporting of results. This does not mean that protocol deviations are not possible, but such changes or additions should be made transparent in the reporting phase.

**Box 2. Key elements of a protocol for a systematic review of observational studies of etiology**

1. Background and rationale
2. Review question(s)
3. Definition of exposures, contrasts, and outcomes
4. Tabulation of potential confounders and biases that could affect study results
5. Study eligibility criteria
6. Literature search for relevant studies
7. Data extraction (study characteristics and results)
8. Assessment of risk of bias and study sensitivity
9. Statistical methods
10. Planned analyses
11. Approach to how the body of evidence will be judged

## Searching for relevant studies

Searching for eligible studies is a process that includes several steps: (i) selection of electronic databases to be searched (e.g., MEDLINE, EMBASE, specialised databases such as Toxicology Literature Online [Toxline], or databases of regulatory authorities); (ii) developing of search strategies and piloting and refining these in collaboration with an information scientist or librarian [28]; (iii) consideration of other approaches, such as citation tracking or scrutinising references of key papers; and (iv) deciding whether to search the grey literature (e.g., conference abstracts, theses, preprints). As many relevant studies may be identified in sources other than electronic databases [29,30], searches that extend beyond the standard electronic databases should be considered.

Identification of observational study designs in literature databases is not straightforward, as the indexing of study types can be inaccurate. Several electronic databases should be searched, as no single database has adequate coverage of all the relevant literature [31]. A bibliographic study concluded that no efficient systematic search strategies exist to identify epidemiologic studies [32]. Compiling a list of key studies that should be identified is helpful to check the sensitivity of the electronic search strategy. As the number of hits from the search may be very large relative to the number of eligible studies, the challenge is to focus the search as much as possible without compromising sensitivity. Summarized Research in Information Retrieval for Human Technology Assessment (HTA) (www.sure--info.org) is a web-based resource that provides guidance on sources to search and on designing search strategies, including search filters to identify observational studies. The incremental value of searching for observational studies in languages other than English has not been evaluated but will depend on the research question. In general, it is prudent to assume that language restrictions could introduce bias. Researchers should search not only for studies on ‘the exposure and outcome’ of interest but have an open mind for studies with negative exposure and outcome controls, ecological and time trend studies of exposure and/or outcome, and papers from basic science.

### Study selection

The search produces bibliographic references with information on authors, titles, journals, etc. However, the unit of interest is the study and not the publication—the same study might have been reported more than once [33], and a single publication can report on multiple studies. First, all identified reports are screened based on title and abstract to remove duplicate publications and articles that are clearly not relevant. This leads to a set of studies for which the full texts are required to determine eligibility and potential overlaps in study populations. Even with clearly defined eligibility criteria, not all decisions will be straightforward. For example, if researchers want to perform a review restricted to children, some studies may have included young adults without providing data for children only. In this case, reviewers may have to decide what proportion of adults is acceptable for a study to be included, or they may attempt to obtain the data on children from the authors.

There is no standard answer to the question of whether study design or methodological quality should guide inclusion of studies [18]. If, for a specific review, a design characteristic is clearly related to high risk of bias and easy to identify (for example, case-control studies of long-term exposures), then such studies could be excluded upfront. An argument for not restricting reviews in this way is that the assessment of risk of bias will often be subjective to some extent, potentially leading to inappropriate exclusions of studies, and that including all studies may lead to important insights when exploring between-study heterogeneity [34].

**Fig 1:**
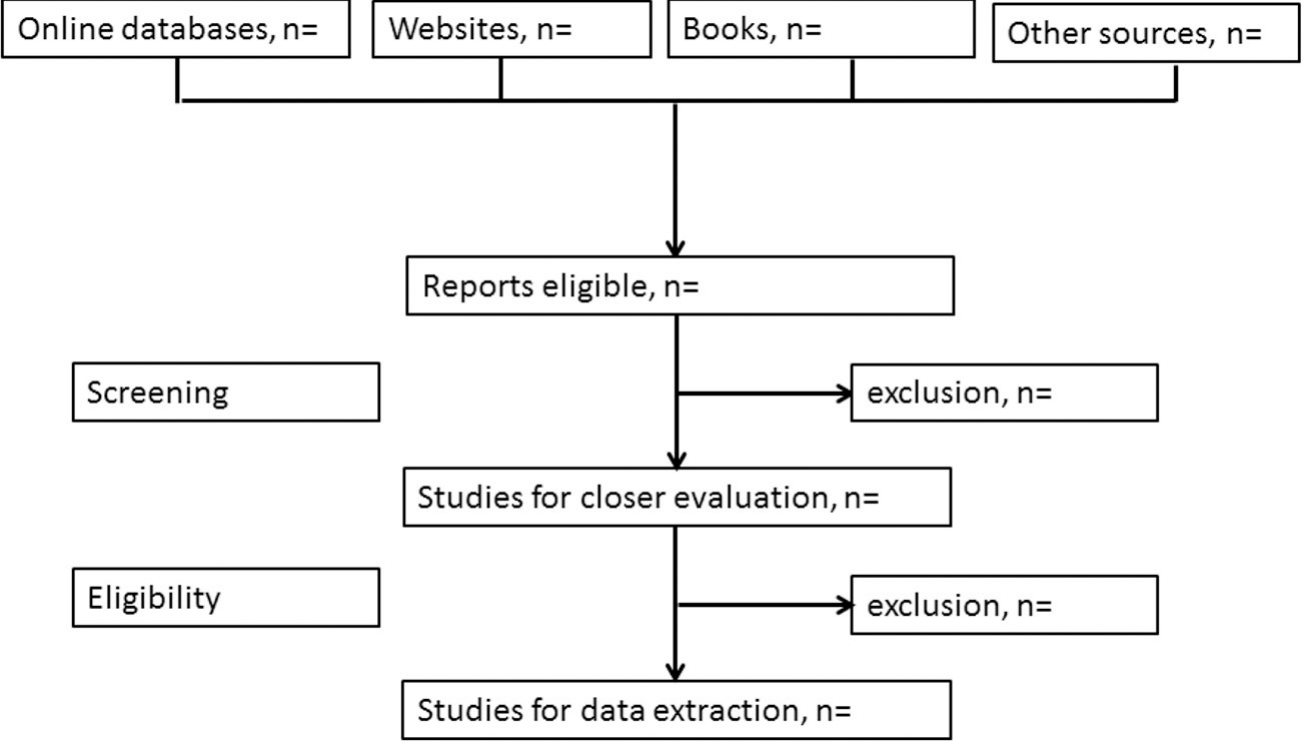
Flow chart of study selection. From [15].

Recording and reporting reasons for exclusions enhances the transparency of the process and informs sensitivity analyses to examine the effect of excluding or including studies. Reviewers should therefore document their decisions throughout the process of study selection and summarise it in a flowchart. Fig 1 gives an example. Dedicated software to support the process of selecting studies may be helpful (see http://systematicreviewtools.com/), including tools using machine learning and text mining to partially automate finding eligible studies and extracting data from articles.

## Data extraction

Article screening, data extraction, and assessments of risk of bias should preferably be done independently by two reviewers to reduce errors and to detect any differences in interpretation between extractors [35,36]. Discrepancies can then be discussed and resolved [37]. Standardised data extraction sheets should be developed for each review, piloted with a few typical studies, refined, and then implemented in generic (for example, EpiData) or preferably dedicated software (for example, Covidence; see http://systematicreviewtools.com). For all included studies, the following core data should be extracted:

1. Bibliographic information
2. Study design
3. Risk of bias assessment
4. Exposure(s) and outcomes, including definitions
5. Characteristics of study participants
6. Numerical results: number of participants per group, number with outcome
7. Effect estimates (adjusted and unadjusted) and their standard errors

Bibliographic information includes the journal or preprint server, publication year, volume and page numbers, and digital object identifier (doi). The definition of the study design should be based on an assessment of what was done, not on how the study was described in the title or indexed in a database [3]. Indexing of observational study designs is often inadequate, and authors may themselves confuse designs. In specialty journals, 30%–50% of studies indexed as 'case-control studies' are not in fact case-control studies [38,39]. Characteristics of study participants and outcomes are extracted separately for exposed and unexposed (cohort studies) or cases and controls (case-control studies).

### Extracting effect estimates and standard errors

Studies will typically report an effect estimate and a measure of precision (confidence interval) or *P* value. The effect estimates may be odds ratios, rate ratios, risk ratios, or risk differences for studies with a dichotomous outcome and the difference in means for continuous outcomes. In general, extraction of the standard error or standard deviation may not be straightforward (a standard error may wrongly be described as standard deviation or vice versa), and sometimes, the standard error needs to be calculated indirectly. S1 Box provides practical guidance.

The confounder-adjusted estimates will be of greatest interest for observational studies, but it is useful to additionally extract the unadjusted estimates or raw data. Comparisons of adjusted and crude estimates allow insights into the importance of confounding. Many studies report effect estimates from different models, adjusted for different sets of confounders. In this situation, meta-analyses of maximally adjusted estimates and minimally adjusted or crude estimates may be performed, as was done in a meta-analysis of insulin-like growth factor and cancer risk [40].

## Assessing quality and bias

The assessment of methodological aspects of studies is a crucial component of any systematic review. Observational studies may yield estimates of associations that deviate from true underlying relationships due to confounding or biases. Meta-analyses of observational studies may therefore produce ‘very precise but equally spurious’ results [41].

The term ‘study quality’ is often used in this context, but it is important to distinguish between quality and risk of bias. The quality of a study will be high if the authors have performed the best possible study. However, a high-quality study may still be at high risk of bias. For example, in a case-control study of lifetime alcohol consumption and endometrial cancer risk, the authors used a state-of-the art population-based design to reduce the risk of selection bias [42] but had to rely on self-reported alcohol intake over a lifetime. It is likely that some women will have underreported their alcohol intake, introducing social desirability bias [43].

The concept of ‘study sensitivity’ [23] refers to the ability of studies to detect a true effect and is more closely related to study quality than bias. If the study is negative, does this really mean that there is evidence for no exposure–outcome association? For example, were the numbers of exposed persons sufficient and the levels and duration of exposure adequate to detect an effect? [23]. Was follow-up long enough to allow for the development of the cancer of interest? Study sensitivity is particularly relevant in occupational and environmental epidemiology but is also of great concern in pharmacoepidemiology in the context of adverse effects of drugs. Reviewers should consider assessing both the risk of bias and study sensitivity in reviews of observational studies.

### Risk of bias in individual studies: Confounding, selection bias, and information bias

Many possible sources of bias exist, and different terms are used to describe them. Bias typically arises either from flawed collection of information or selection of participants into the study so that an association is found that deviates from the true value. Typically, bias is introduced during the design or implementation of a study and cannot be remedied later.

In contrast to bias, confounding produces associations that are real but not causal because some other, unaccounted factor is associated with both exposure and outcome (Box 3). Time-dependent confounding is a special case of confounding (S2 Box). Confounding can be adjusted for in the analysis if the relevant confounding variables have been well measured, but some residual confounding may remain after adjustment (Box 4). Confounding is often confused with selection bias. In particular, the situation in which comparison groups differ with respect to an important prognostic variable is often described as selection bias. The term selection bias should, however, be used only for the situation in which participants, their follow-up time, or outcome events are selected into a study or analysis in a way that leads to a biased association between exposure and outcome. Directed acyclic graphs (DAGs) are useful to clarify the structures of confounding and selection bias (see Box 3) [44]. Another important category of bias is information bias, in which systematic differences in the accuracy of exposure or outcome data may lead to differential misclassification of individuals regarding exposures or outcomes. Bias needs to be distinguished from random error, a deviation from a true value caused by chance variation in the data. Finally, it is important to note that confounding and selection bias refer to biases that are internal to the study (‘internal validity’) and not to issues of generalisability or applicability (‘external validity’) [20]. How should the risk of bias in observational studies best be assessed? A review identified more than 80 tools for assessing risk of bias in nonrandomised studies [45]. The reviewers concluded that there is no ‘single obvious candidate tool for assessing quality of observational epidemiological studies’. This is not surprising considering the large heterogeneity in study designs, contexts, and research questions in observational research. We believe that the quest for a ‘one size fits all’ approach is misguided; rather, a set of criteria should be developed for each observational systematic review and meta-analysis, guided by the general principles outlined below.

**Box 3. The causal structures of confounding and selection bias**

**Fig 2:**
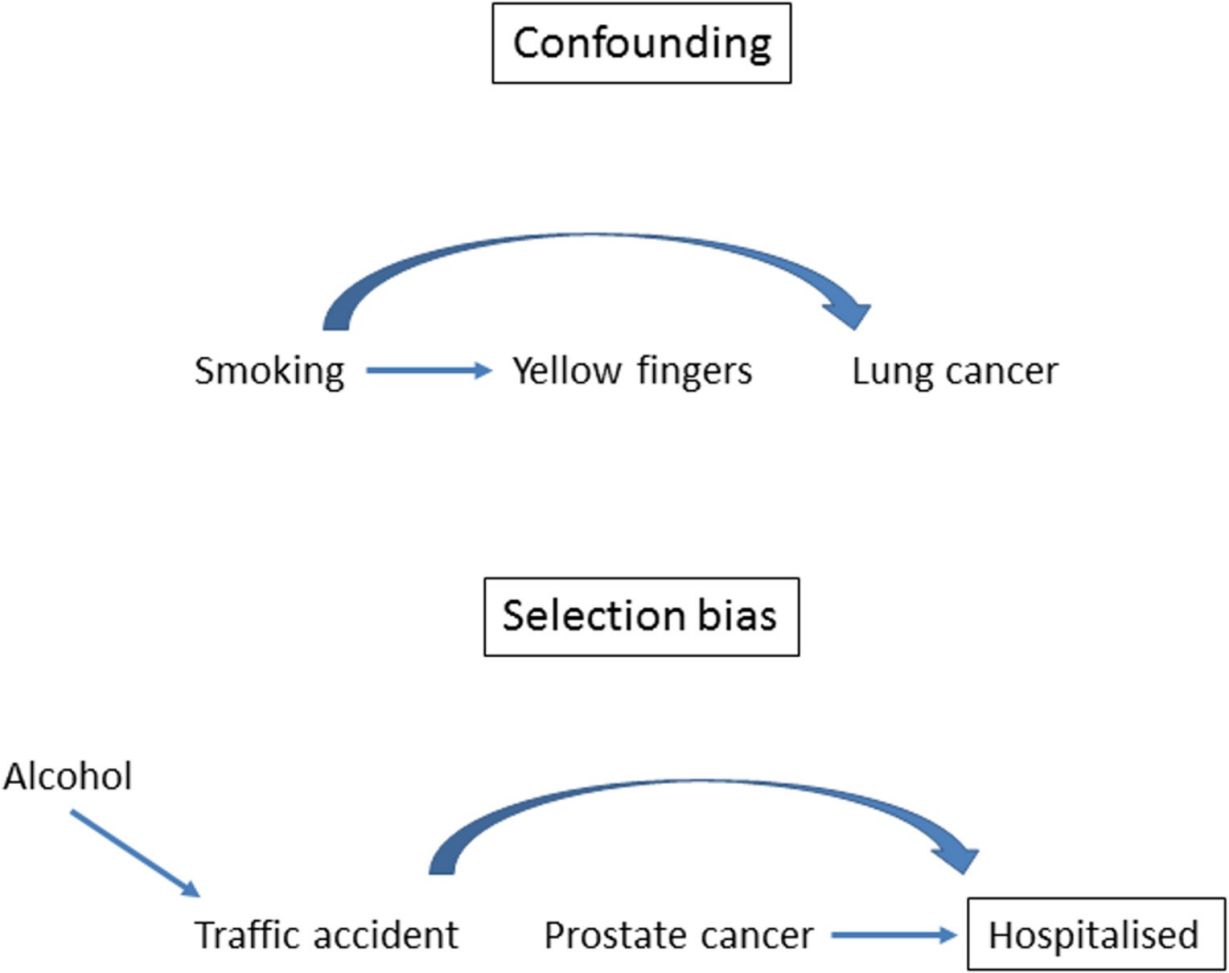
The causal structures of confounding and selection bias.

A directed acyclic graph (DAG) consists of (measured and unmeasured) variables and arrows. They are useful to depict causal structures: arrows are interpreted as causal effects of one variable on another [37]. Common causes of exposure and outcome confound the association between exposure and outcome. For example, as shown in the DAG in Fig 2, the association between yellow fingers and lung cancer is confounded by smoking. The association is spurious, i.e., it is entirely explained by smoking.

Selection bias occurs if the probability of inclusion into the study depends both on the exposure and the outcome. In a hospital-based case-control study, inclusion into the study naturally depends on being admitted to the hospital; it is conditional on hospitalisation. For example, in a case-control study of alcohol and prostate cancer, the inclusion of controls hospitalised because of injuries suffered in traffic accidents will introduce an association between alcohol consumption and prostate cancer because alcohol is a cause of traffic accidents (Fig 2 for a graphical display). In general, conditioning on common effects of exposure and outcome means that the probability of inclusion depends on the exposure and outcome, which leads to selection bias. This structure applies to many biases, including nonresponse bias, missing data bias, volunteer bias or health worker bias, or bias due to loss of follow-up in cohort studies [44]. All of these biases have the causal structure of selection bias.

**Box 4. Approaches to deal with confounding in observational studies**

Statistical adjustment in the analysis

Statistical adjustment for confounding can be performed using standard regression techniques (for example, Cox or logistic regression). All of these models, including more advanced techniques such as propensity scores [46] or inverse probability weighting, rely on the assumption of no unmeasured confounding for the validity of the effect estimate. This assumption is often unlikely to hold in practice because some confounding factors may not have been assessed or have not been measured precisely. Residual confounding may therefore persist after adjustment, which should be taken into account when interpreting combined estimates from meta-analysis of observational studies.

Matching

Matching is an intuitive approach to control for confounding at the design stage of a study, particularly in case-control research [47]. The choice of the variables, exact approach to matching, and the statistical analysis are complex and need careful consideration. Bias may be introduced, for example, when matching for variables that are on the causal pathway from exposure to disease [3]. Matching is particularly relevant in situations in which the distribution of important confounders differs substantially between cases and unmatched controls [48].

Instrumental variable methods

Instrumental variables may be useful to control for confounding [49]. An instrument is an external variable that is associated with the exposure of interest but ideally is not associated with the outcome variable other than through its effect on the exposure. In essence, instrumental variables circumvent the problem of unmeasured confounding. For example, mendelian randomisation studies make use of common genetic polymorphisms (for example, the rs1229984 variant in the alcohol dehydrogenase 1B gene) that influence levels of modifiable exposures (alcohol intake [50]). As with any other instrumental variable analysis, the validity of mendelian randomisation depends critically on the absence of a relationship between the instrument (genes) and the outcome (for example, cardiovascular disease) [5].

Negative controls

The use of ‘negative controls’ for outcomes or exposures can be helpful to assess the likely presence of unmeasured or residual confounding in observational research [51]. The rationale is to examine an association that cannot plausibly be produced by the hypothesised causal mechanism but may be generated by the same sources of bias or confounding as the association of interest. For example, it has been hypothesised that smoking increases the risk of depression and suicide because of effects on serotonin and monoamine oxidase levels [52]. However, in large cohort studies, smoking is also positively associated with the risk of being murdered—the negative, biologically implausible outcome control [53]. Similarly, in cohort studies, vaccination against influenza not only protected against hospitalisation for pneumonia but also against hospitalisation for injury or trauma [46], indicating that the beneficial effect on pneumonia may have been overestimated. Negative exposure controls are usefully introduced in questionnaires to gauge possible recall bias in case-control studies. A study of the association between multiple sclerosis (MS) and childhood infections included questions on other childhood events not plausibly associated with MS, such as fractures and tonsillectomy [54].

### General principles

When assessing the risk of bias, seven general principles are relevant, based on theoretical considerations, empirical work, and the experience with assessing risk of bias in RCTs and other studies [55–57].

#### 1. The relevant domains of bias should be defined separately for each review question and for different study designs

Relevant domains of risk of bias that should be considered include (i) bias due to (time-dependent) confounding, (ii) bias in selection of participants into the study (selection bias), (iii) bias in measurement of exposures or outcomes (information bias), (iv) bias due to missing data (selection bias), and (v) bias in selection of studies or reported outcomes (selection bias) [56]. The risk of bias should be assessed for each domain and, if required, for different outcomes. The focus should be on bias. For example, whether or not a sample size calculation was performed or ethical approval was obtained does not affect the risk of bias.

#### 2. The risk of bias should be assessed qualitatively

For each study and bias domain, the risk of bias should be assessed in qualitative categories, for example, as ‘low risk’, ‘moderate risk’, or ‘high risk’. These categories and the criteria used to define them should be described in the paper. Quantitative assessments by assigning points should be avoided (see also point 6).

#### 3. Signalling questions may be useful

Within each bias domain, simple signalling questions may be useful to facilitate judgments about the risk of bias (Table 1). A comprehensive list of signalling questions has recently been compiled by the developers of the Cochrane risk of bias assessment tool for nonrandomised studies of interventions (ROBINS-I) [56], and a similar tool is in development for nonrandomised studies of exposures [58]. These lists and tools will be useful, but reviewers should think about further questions that may be relevant in the context of their review. Cooper and colleagues compiled a list of questions relating to study sensitivity [23].

**Table 1:** Signalling questions for different bias domains.

*Bias domain*	*Study design*	*Signalling question*
**Confounding**	Any	What are the important variables that might confound the effect of the exposure?
	Any	Were these variables measured with precision and at appropriate points in time?
	Any	Did the authors use an appropriate analysis method or design that adjusted for all of the important confounding variables?
**Selection bias**	Cohort studies/cross-sectional studies	Was selection into the study unrelated to both the exposure and outcomes?
	Cohort studies/cross-sectional studies	Were the reasons for missing data unrelated to the exposure and outcomes?
	Case-control studies	Were the controls sampled from the population that gave rise to the cases?
	Case-control studies	Were the reasons for missing data related to case or control status?
**Information bias**	Cohort studies/cross-sectional studies	Were outcome assessors unaware of the exposure status of study participants?
	Cohort studies/cross-sectional studies	Were the methods of outcome assessment comparable across exposure groups?
	Case-control studies	Was the definition of case status/control status applied without knowledge of exposure status?
	Case-control studies	Was data collection on exposure status unaffected by knowledge of the outcome or risk of the outcome?

#### 4. Separate assessments may have to be made for different outcomes

The risk of bias will often differ across different outcomes. For example, bias in the ascertainment of death from all causes is much less likely than for a subjective outcome, such as quality of life or pain, or for an outcome that relies on clinical judgment, such as pneumonia.

#### 5. Assessments should be documented

It is good practice to copy and archive the text from the article on which an assessment regarding the risk of bias is based. Such documentation increases transparency, facilitates discussion in case of disagreement, and allows for replication of assessments.

#### 6. Summary scores should be avoided

Summary scores involve weighting of bias domains; typically, each item in a score is weighted equally (0 or 1 point), but the importance of a bias will depend on the context, and one bias may be more important than another [59,60]. The situation is made worse if the scale includes items that are not consistently related to bias. For example, the Newcastle-Ottawa Scale includes quality items of questionable validity, such as comparable nonresponse among cases and controls [61]. Rather than calculating summary scores, a conservative approach classifies the study at the level of risk of bias corresponding to the highest risk identified for individual domains.

#### 7. Thinking about a hypothetical, unbiased trial may be helpful

It may useful, as a thought experiment, to think of a hypothetical RCT that would answer the review question posed in the systematic review [56]. Such a trial will often be unfeasible and unethical, but the thought experiment may help to sharpen the review question and clarify the potential biases in the observational studies. S3 Box gives an example.

### Reporting biases, *P* hacking, and analytic choices

An important source of bias that may undermine conclusions from any systematic review or meta-analysis is the selective publication of studies. It is known that studies with ‘positive’ results (i.e., statistically significant effects) are more likely to be published than negative studies, introducing a distorted overall picture of an association. There is robust evidence of publication bias and other reporting biases for RCTs [62,63]: positive trials are more likely to be published, to be published quickly, to be published more than once, and to be cited, making it more likely that they will be included in systematic reviews.

Selective publication of results may also be a problem within studies, when many different exposures and outcomes were examined. If only the statistically significant associations are fully analysed, written up, and published, the results of systematic reviews will be distorted. A related issue arises when the selection of the study population or statistical model is chosen based on the *P* value (‘*P* hacking’) [64].

### How to deal with risk of bias?

Results from the risk of bias analysis should be presented in a transparent way, tabulating risk of bias elements for each included study. An important consideration is how to deal with studies at high risk of bias. If the aim is to present the best available evidence on the efficacy of a medical intervention, the review is often restricted to studies at low risk of bias. For systematic reviews of observational studies of etiology, we generally advise against excluding studies based on risk of bias assessments. Including all studies and exploring the impact of the risk of different biases and of study sensitivity on the results in stratified or regression analyses will often provide additional insights, as discussed below.

## Exploring and exploiting heterogeneity

The studies included in a systematic review will generally vary with respect to design, study populations, and risk of bias [1]. Mapping of heterogeneity between studies [65] may not only provide a useful overview but also help decide whether or not to combine studies statistically in a meta-analysis. Such diversity may provide opportunities for additional insights and can explicitly be exploited [66]. For example, the association of *Mycobacterium avium* subspecies *paratuberculosis* (MAP) with Crohn disease was examined in a review of case-control studies that compared cases of Crohn disease with controls free of inflammatory bowel disease or with ulcerative colitis patients [67]. The association was strong for both comparisons, indicating that the association with MAP is specific to Crohn disease and not a general (epi)phenomenon in inflammatory bowel disease (see also Box 4 on negative controls).

Diversity in study settings also may provide insights. Lifestyle factors such as smoking, physical activity, sexual behaviour, or diet are exposures of interest in many observational studies, but they are highly correlated in Western societies. Their independent effects, for example, on cancer risk, are therefore difficult to disentangle. The inclusion of studies in special populations, for example, defined by religion or geographical regions with different lifestyle patterns, may therefore help understand (residual) confounding. Similarly, studies that measured exposures and confounding factors more or less precisely, used different methods to adjust for confounding variables (Box 4), or were generally at higher or lower risk of bias will be valuable in this context. For example, a meta-analysis showed that the relationship between induced abortion and breast cancer was evident in case-control studies but not in cohort studies [68]: the association observed in case-control studies was probably due to recall bias.

The thoughtful exploitation of sources of heterogeneity at the design stage or exploration in the analysis are therefore an important part of systematic reviews and meta-analyses of observational studies of etiology. Analyses should either be prespecified in the study protocol or interpreted in the spirit of exploratory data analyses. Exploration of heterogeneity starts with the visual inspection of forest plots and funnel plots. Statistical techniques include subgroup analyses and metaregression, as discussed below.

## Meta-analysis: To pool or not to pool?

After careful examination of risk of bias and other sources of heterogeneity, reviewers must consider whether statistically combining effect estimates is appropriate for all studies, for a subgroup of studies, or not appropriate at all. Authors provide different reasons for not pooling data [69], and different opinions exist on how to approach this question [18]. Considerations for or against pooling should be based on judgments regarding study diversity, sensitivity, and risk of bias rather than solely on statistical measures of heterogeneity (see below) for two reasons. First, in the absence of statistical heterogeneity, combining results from biased studies will produce equally biased combined estimates with narrow confidence intervals that may wrongly be interpreted as definitive evidence. The inclusion of studies at high risk of bias will often, but not always, introduce heterogeneity. For example, the protective effect of a diet rich in beta-carotene on cardiovascular mortality shown in observational studies was very consistent across studies. However, randomised trials of beta-carotene supplementation did not show any benefit, making it likely that the results of observational studies were confounded to a similar extent by other aspects of a healthy diet and lifestyle [41]. Second, even in the presence of statistical heterogeneity, combining studies may be appropriate if studies are at low risk of bias and results are qualitatively consistent, indicating some degree of benefit or risk. If authors decide not to provide one overall pooled estimate, stratified meta-analyses (by study design or population) may be considered. Be mindful that a systematic review that documents heterogeneity and risk of bias will still provide a valuable contribution even without a formal meta-analysis.

## Statistical analysis

### Fixed- versus random-effect models in the context of observational studies

Once the decision has been made that (some) studies can be combined in a meta-analysis, reviewers need to decide whether to use a fixed-effect or a random-effects model or both. These models have been described extensively [70]. In short, fixed-effect analyses assume that all studies estimate the same underlying effect and that differences between effect estimates are due to sampling variation. In contrast, random-effects models assume that underlying true effects vary between studies. In the presence of statistical heterogeneity, effect estimates will differ because smaller studies receive more weight in the random-effects than in the fixed-effect model, and the random-effects confidence interval will be wider because it additionally incorporates the between-study variation. In the absence of statistical heterogeneity, results from random- and fixed-effect models will be identical.

In observational studies, population characteristics and exposure or outcome definitions will likely differ across studies. The assumption that all these studies estimate the same underlying effect is rarely justified, and using a random-effects model for combining observational studies therefore seems reasonable [18]. An important consideration in this context is the question of whether, for a given research question, smaller or larger studies are at greater risk of bias. In clinical research, large multicentre trials tend to be at lower risk of bias than small single-centre studies, supporting the use of fixed-effect models. The opposite may be the case in observational etiological research, for which smaller studies may have collected better data on exposures and confounders. The model to be used should be specified in advance, but presenting results from both models in a sensitivity analysis may be informative. Of note, although random-effects models allow for between-study heterogeneity, they do not help to understand the sources of heterogeneity [71].

### Statistical measures of between study heterogeneity

Methods to assess statistical heterogeneity include the *I*^*2*^ statistics and Cochrane’s Q test for heterogeneity. The Q test assesses whether variation between effect estimates is likely due to chance alone; the *I*^*2*^ statistic quantifies the amount of variation between studies that cannot be attributed to chance [72]. These measures should be interpreted with caution: the *I*^*2*^ statistic is captured with uncertainty [73], and Cochrane’s Q test has limited power to detect heterogeneity when the number of included studies is low [74]. Since the number of studies included in a review of observational studies is typically 10 to 20 [1], statistical power will generally be low. Moreover, the statistical verdict on presence or absence of heterogeneity does not need to coincide with the reviewers’ judgment on presence or absence of study diversity or risk of bias. It might be that important study diversity does not translate into statistical heterogeneity.

### Funnel plot (a)symmetry

A funnel plot is a graphical tool to investigate whether estimates from smaller studies differ from those of larger studies. Effect sizes are plotted against the standard error or precision of the estimate (which relate to study size) [75]. If estimates from different studies differ only because of random variation, then they will scatter symmetrically around a central value, with variation decreasing as precision increases. The plot will thus resemble an inverted funnel. Asymmetry of the funnel plot means that there is an association between study size and effect estimates or a ‘small study effect’ [76], with smaller studies typically showing larger effects. This may be due to several reasons, including true heterogeneity (i.e., smaller studies differ from larger ones in terms of study population, exposure levels, etc.), selection bias (i.e., the selective publication of small studies showing an effect), bias in design or analysis, or chance [77,78]. Asymmetry should not be equated with publication bias. Particularly in the context of observational studies, there are many other sources of heterogeneity and funnel plot asymmetry.

### Metaregression

Metaregression is used to investigate whether study characteristics are associated with the magnitude of effects and whether specific study characteristics can explain (some of) the observed statistical heterogeneity. The presence of heterogeneity motivates metaregression analyses, and random-effects metaregression should therefore always be used. The use of fixed-effect metaregression is conceptually nonsensical and yields a high rate of false-positive results [79].

**Fig 3:**
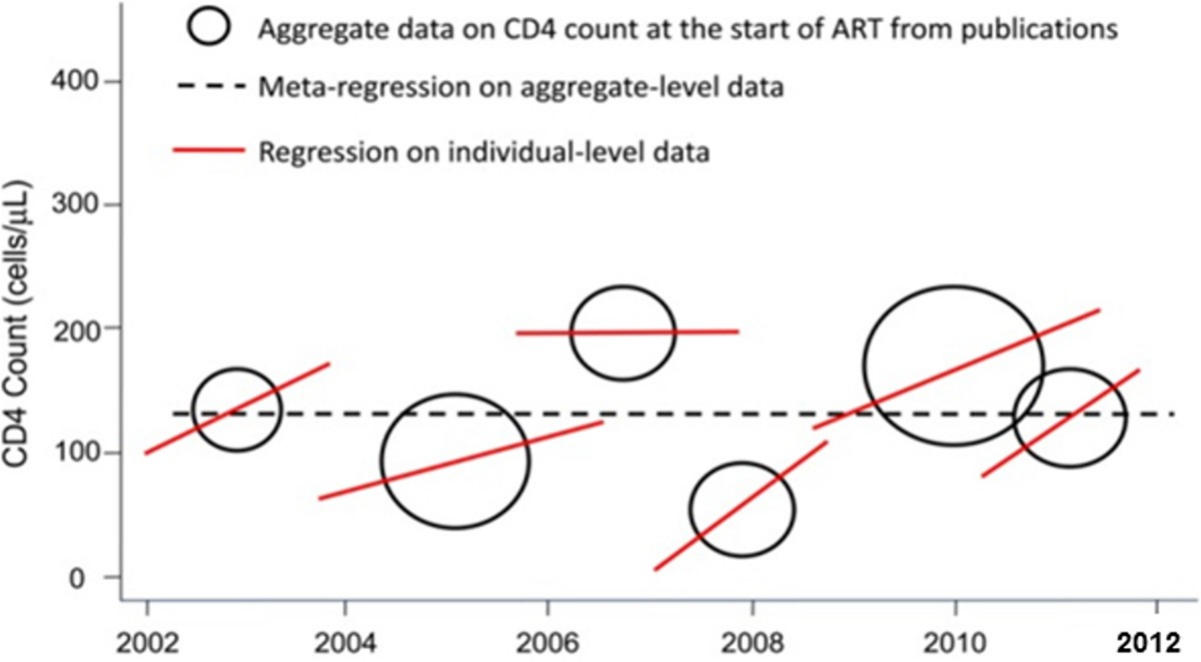
Illustration of the ecological fallacy. Hypothetical example of aggregate and individual level CD4 cell count data at the start of ART. Adapted from [83]. ART, antiretroviral therapy.

Variables included in a metaregression model may be study features, such as study design, year of publication, or risk of bias; or characteristics of the people included in the different studies, such as age, sex, or disease stage. These variables are potential effect modifiers. For example, the risks of smoking decrease with advanced age. Only a few variables should be included in a metaregression analysis (about one variable per 10 studies), and they should be prespecified to minimise the risk of false-positive results [80]. In multivariable metaregression, the model presents mutually adjusted estimates, and permutation tests to adjust for multiple testing can be considered [80]. When including characteristics of study participants, note that associations observed at the study level may not reflect those at the individual level—the so-called ecological fallacy [81]. This phenomenon is illustrated in Fig 3 for trends in the CD4 positive lymphocyte count in HIV-positive patients starting antiretroviral therapy (ART): in five of the six studies, the CD4 cell count at the start of ART increased over time, which was not shown in metaregression analysis at the study level. A graphical display of the metaregression is informative [82]. Such a graph shows for each study the outcome (e.g., a relative risk or a risk difference) on the y-axis, the explanatory variable on the x-axis, and the regression line that shows the association between two variables. In a metaregression graph, the weight of the studies is preferably shown by ‘bubbles’ around the effect estimates, with larger bubbles relating to studies with more weight in the analysis (Fig 3 provides a schematic example).

### Combining different metrics

Meta-analyses depend on how data are presented in individual articles. Especially in the context of observational studies, researchers may face the problem that different metrics are used for the same exposure–outcome association, depending on study design or statistical models used. Consider the association between smoking (exposure) and blood pressure (outcome). In cohort studies, hazard ratios, incidence rate ratios, risk ratios, or odds ratios can be estimated when the outcome is dichotomized, whereas mean difference or standardised mean difference may be reported if this is not the case.

How can different metrics be combined in meta-analysis? There are two issues to consider, one conceptual, one more technical. When studies report different ratio metrics, for example, hazard ratios, risk ratios, or odds ratios, they may be combined ignoring the differences in metrics. This may be appropriate depending on which study designs were included (cohort studies or case-control studies) and how participants were sampled in case-control studies [84,85]. As a general rule, the different ratio metrics can be combined if the outcome under study is rare (<5%), which is often the case in etiologic studies. If the outcome is not rare, researchers must be more careful because the odds ratio will substantially overestimate the relative risk. This property of the odds ratio is a reflection of the fact that for non-rare outcomes, the odds is larger than the risk (for example, if the risk is 0.8, the corresponding odds is 4).

It is also possible to combine relative risks with other metrics like standardised mean differences or correlation coefficients. This requires transformation from an odds ratio in a standardised mean difference (or vice versa) [86] or an odds ratio in a correlation coefficient [87,88]. For example, in a meta-analysis of the association between fibrinogen levels on postoperative blood loss, studies reported odds ratios, regression coefficients, correlation coefficients, and/or *P* values [89]. All effect measures were transformed into correlation coefficients and subsequently combined in a meta-analysis [89].

### Dose-response meta-analysis

In many epidemiologic studies, several levels of exposure are compared. For example, the effect of blood glucose on cardiovascular outcomes can be studied across several groups of glucose levels, using one category as reference. However, different studies may report different categories of the exposure variable (tertiles, quartiles, or quintiles). One approach is to meta-analyse the estimates by comparing the lowest and highest category. This is not recommended because the meaning of lowest versus highest differs across studies. A more sophisticated approach is to model the association between the exposure and outcome to estimate the increase (or decrease) in risk associated with one unit (or other meaningful incremental) increase in exposure. See references for technical details [90,91]. For example, a meta-analysis of the association between Homeostasis Model Assessment Insulin Resistance (HOMA-IR) and cardiovascular events used dose-response modelling to estimate that the cardiovascular risk increased by 46% per one standard deviation increase in HOMA-IR [24].

## Interpretation and discussion of results

Reviewers should discuss their results in a balanced way: many of the included studies might be far from perfect, even if the overall estimate comes with a narrow confidence interval [41], and researchers should keep in mind that statistical significance is not an indicator of whether a true relation exists or not. Big numbers cannot compensate for bias. If included studies have a low risk of bias and heterogeneity does not seem large, researchers may conclude that the main results provide reasonably valid estimates. On the other hand, if many studies are at high risk of bias, researchers should conclude that the true effect remains uncertain. The Grades of Recommendation, Assessment, Development, and Evaluation (GRADE) system can be helpful to formally judge ‘the extent of our confidence that the estimates of an effect are adequate to support a particular decision or recommendation’ [92], taking into account study design, risk of bias, degree of inconsistency, imprecision and indirectness (applicability) of results, and reporting bias [93].

One or a few studies might suffice to demonstrate that a relevant bias likely exists and that all other studies suffer from it. For example, many cohort studies have shown that higher C-reactive protein (CRP) levels are associated with cardiovascular risk. However, other cardiovascular risk factors, including smoking, obesity, and physical activity, are associated with higher CRP levels, and these may confound the association with cardiovascular disease levels [94]. No association was seen in mendelian randomisation studies [94], which used genetic variants that are related to CRP levels but independent of the behavioural or environmental risk factors that confound the association in epidemiological studies [95] (see Box 1). Mendelian randomisation studies and classic cohort studies in fact estimate different ‘effects’: lifelong exposure in mendelian randomisation versus exposure from a certain (often not well-defined) time-point onward. It should be noted that when mendelian randomisation studies include participants later in life, selection bias may occur [96]. Evidence from mendelian randomisation studies, if available, should always be taken into account when interpreting the results from systematic reviews of epidemiological studies. A useful guide for reading mendelian randomisation studies has recently been published [97].

When assessing causality, integration of different sources of evidence (e.g., ecological studies, basic research on mechanisms) may facilitate a final judgement; trying to obtain an integrated verdict based on results from different analytic or epidemiologic design approaches, for which each approach has different and preferably unrelated sources of potential bias, is called triangulation [98]. If different approaches all point to the same conclusion, this strengthens confidence that the finding may be causal [98]. For example, in the discussion on smoking and lung cancer, time trends in lung cancer were an important argument against the hypothesis that an inherited trait would cause lung cancer as well as smoking [99]. Discussing competing explanations systematically will add value to the interpretation of the results [100]. Especially in the field of toxicology, mechanistic evidence plays an important role in causal inference, and systematic review of this literature is encouraged [17]. Clearly, understanding pathways requires more than quickly searching for a few articles that support the hypothesis ('cherry picking') [17]. Systematic reviews on insulin-like growth factor or adiposity and cancer risk took laboratory, animal, and human evidence into account to judge the plausibility of different mechanisms [40,101].

Finally, the importance of the results in terms of clinical and public health relevance should be discussed. The identification of likely causes does not necessarily translate into recommendations for interventions [102]. For example, based on epidemiological and other evidence, obesity probably increases the risk of several cancers [101,103], but this does not mean that losing weight will reduce cancer risk. Obesity may have exerted its detrimental effect, and different interventions to reduce obesity have different effects on cancer risk [104].

An important strength of systematic reviews is that they generate a clear overview of the field and identify the gaps in the evidence base and the type of further research needed. The usual statement that 'more research is needed' can thus be replaced by detailed recommendations of specific studies. Furthermore, having assessed the strengths and limitations of many studies, reviewers will be in an excellent position to name the pitfalls that need to be avoided when thinking about future research.

## Supporting information

S1 Box (DOCX)

S2 Box (DOCX)

S3 Box (DOCX)

## Abbreviations

ART: antiretroviral therapy
COSMOS-E: Conducting Systematic Reviews and Meta-Analyses of Observational Studies of Etiology
CRP: C-reactive protein
DAG: directed acyclic graph
doi: digital object identifier
EMBASE: Excerpta Medical database
GRADE: Grades of Recommendation, Assessment, Development, and Evaluation
HOMA-IR: Homeostasis Model Assessment Insulin Resistance
HTA: Human Technology Assessment
MAP: *Mycobacterium avium* subspecies *paratuberculosis*
MEDLINE: Medical Literature Analysis and Retrieval System Online
MRI: magnetic resonance imaging
MS: multiple sclerosis
PECO: Population, Exposure, Control, and Outcome(s)
PICO: Population, Intervention, Control, and Outcome(s)
PROSPERO: International Prospective Register of Systematic Reviews
RCT: randomised controlled trial
ROBINS-I: risk of bias assessment tool for nonrandomised studies of interventions
Toxline: Toxicology Literature Online
