# Supplementary material for: COSMOS-E: Guidance on conducting systematic reviews and meta-analyses of observational studies of etiology
Source: PLoS Med. 2019 Feb 21;16(2):e1002742. doi: 10.1371/journal.pmed.1002742 (PMC6383865; doi:10.1371/journal.pmed.1002742)
Supplement: S1 Box — (DOCX) [file pmed.1002742.s001.docx]

| **S1 Box. Approaches to extracting standard errors and standard deviations.** |
| --- |
| Reports of observational studies often do not include the standard errors for the estimates of interest. Below we give some guidance on how to obtain standard errors from other statistics, such as confidence intervals or p values.  Only confidence intervals are reported: Confidence intervals can be used to obtain standard errors according to the following formulae. For ratio measures, all calculations should be done on the logarithmic scale, as this formula works for symmetric confidence intervals:  $\text{Standard}\text{ }\text{error }\text{= }\frac{\text{Effect}\text{ }\text{estimate }-\text{ Lower}\text{ }\text{limit}\text{ }\text{of confidence}\text{ }\text{interval}}{\text{1.96}}$  Only P values are reported: In this instance the P value can be transformed to a z-score which is used to derive the standard error[1]:  $z=-0.862+\surd[0.743-2.404 \times\text{log} (\text{P})]$  $\text{Standard}\text{ }\text{error} = \frac{\text{Effect}\text{ }\text{estimate}}{z}$  Only significance levels are reported: If only significance levels are reported (P< or > 0.05), standard errors cannot be calculated. If, however, the p-value is reported as <0.05, a conservative approach is to set P = 0.0499 (boundary P value) and obtain a standard error using the formula above.  No confidence intervals or p-values reported: When the effect measure of the study is a ratio measure one option is to derive the standard error from the raw 2x2 data. The assumption is that confounding does not largely influence the standard error. For studies with continuous outcomes, but missing standard deviation, an option is to 'borrow' the mean standard deviation from other articles, if these studies have similar clinical patients included . |

## References

1. Altman DG, Bland JM. How to obtain the confidence interval from a P value. BMJ (Clinical research ed). 2011;343:d2090.
