# Supplementary material for: COSMOS-E: Guidance on conducting systematic reviews and meta-analyses of observational studies of etiology
Source: PLoS Med. 2019 Feb 21;16(2):e1002742. doi: 10.1371/journal.pmed.1002742 (PMC6383865; doi:10.1371/journal.pmed.1002742)
Supplement: S2 Box — (DOCX) [file pmed.1002742.s002.docx]

| **S2 Box. Time-related confounding and bias.** |
| --- |
| Time-varying (or time-dependent) confounding should be considered whenever follow-up time in cohort studies whenever exposure status changes over follow-up time. Time-varying confounding has been well documented in pharmacoepidemiology. For example, in HIV-infected patients the number of CD4 receptor positive lymphocytes (CD4 count) is a time-dependent confounder of the effect of antiretroviral therapy. A low CD4 count is an important risk factor for progression to AIDS and death and patients with lower counts are more likely to be treated. CD4 count is in turn affected by therapy and thus intermediate on the causal pathway from antiretroviral treatment to death. In this situation, which can be seen as a type of confounding by indication, standard types of analyses, for example Cox proportional hazard models adjusted for baseline CD4 counts or time-updated CD4 counts, will give biased estimates. Special methods are required in this situation [1, 2].  Combining studies that do account for time-related biases with studies that do not can lead to misleading results. For example, 13 observational studies were found evaluating the association between metformin use among individuals with diabetes and cancer risk [3]. Nine studies that were subject to time-related biases with as a consequence implausibly large effects, with reductions in cancer risk with metformin ranging from 20 to 94%. Three studies that avoided these biases reported no effect of metformin use on cancer incidence. |

## References

1. Cole SR, Hernan MA. Constructing inverse probability weights for marginal structural models. American journal of epidemiology. 2008;168(6):656-64.

2. Taubman SL, Robins JM, Mittleman MA, Hernan MA. Intervening on risk factors for coronary heart disease: an application of the parametric g-formula. International journal of epidemiology. 2009;38(6):1599-611.

3. Suissa S, Azoulay L. Metformin and the risk of cancer: time-related biases in observational studies. Diabetes care. 2012;35(12):2665-73.
