# Supplementary material for: COSMOS-E: Guidance on conducting systematic reviews and meta-analyses of observational studies of etiology
Source: PLoS Med. 2019 Feb 21;16(2):e1002742. doi: 10.1371/journal.pmed.1002742 (PMC6383865; doi:10.1371/journal.pmed.1002742)
Supplement: S3 Box — (DOCX) [file pmed.1002742.s003.docx]

| **S3 Box . Thinking about a hypothetical, unbiased trial.** |
| --- |
| A reviewer aims to examine the evidence on a potential causal effect of smoking on the risk of dementia and considers what a hypothetical RCT addressing this question would look like. For example, 70-years old lifetime non-smokers could be randomized to smoking one pack a day or to continue non-smoking. The rate of incident dementia over 10 years could then be compared between the two groups. Such an RCT will give us an unbiased estimate of the effect of smoking on the risk of dementia among those starting smoking at age 70.  Cohort studies, in contrast, will typically compare elderly people who have had the habit of smoking for many years with life-time non-smokers. The comparison in the observational study will be affected by selection bias due to the competing risk of death: smoking substantially increases the risk of death. Smokers included in the cohort study will have survived to age 70 years and are not comparable to lifetime non-smokers of the same age. Indeed, it has been shown that the association of smoking with dementia becomes weaker as the age of the study population increases [1, 2].  Similar issues can arise in observational studies of drug interventions which are commonly based on prevalent users, whereas randomized clinical trials typically examine incident (‘new’) users.[3] Whether or not the use of prevalent exposures really is a potential problem should be decided case by case. For example, for the study of smoking and lung cancer it is not a problem to enroll prevalent smokers due to the long latency. Indeed, studies of smoking and lung cancer would be impossible to set up under the requirement of ‘incident users’ [4].  Back to our reviewer and the potential association between smoking and dementia: after pondering the issue, she may decide to include cohort studies of all ages, and to stratify analyses by the age of the study population to assess the importance of survivor bias. |

## References

1. Hernan MA, Alonso A, Logroscino G. Cigarette smoking and dementia: potential selection bias in the elderly. Epidemiology. 2008;19(3):448-50.

2. Euser SM, Schram MT, Hofman A, Westendorp RG, Breteler MM. Measuring cognitive function with age: the influence of selection by health and survival. Epidemiology. 2008;19(3):440-7.

3. Danaei G, Tavakkoli M, Hernan MA. Bias in observational studies of prevalent users: lessons for comparative effectiveness research from a meta-analysis of statins. American journal of epidemiology. 2012;175(4):250-62.

4. Vandenbroucke J, Pearce N. Point: incident exposures, prevalent exposures, and causal inference: does limiting studies to persons who are followed from first exposure onward damage epidemiology? American journal of epidemiology. 2015;182(10):826-33.
